# Maternal and Neonatal Vulnerabilities Associated with Abnormal Outcomes in Newborn Hearing Screening: A Focus on Adolescent Mothers

**DOI:** 10.3390/audiolres16010014

**Published:** 2026-01-20

**Authors:** Mirela Mătăsaru, Elena Niculet, Emil Anton, Ancuța Lupu, Oana Ramona Roșca, Doina Carina Voinescu, Mădălina Nicoleta Matei, Alina Pleșea-Condratovici, Camer Salim, Silvia Fotea

**Affiliations:** 1 “Sf. Apostol Andrei” County Emergency Clinical Hospital, 177 Brăilei Street, 800578 Galati, Romania; 2 Doctoral School, Faculty of Medicine and Pharmacy, “Dunărea de Jos” University of Galati, 800008 Galati, Romania; 3 Faculty of Medicine and Pharmacy, “Dunărea de Jos” University of Galati, 35 Al. I. Cuza Street, 800008 Galati, Romania; helena_badiu@yahoo.com (E.N.); alina.plesea@ugal.ro (A.P.C.);; 4 Department of Morphological and Functional Sciences, Faculty of Medicine and Pharmacy, “Dunărea de Jos” University of Galati, 800010 Galati, Romania; 5 Multidisciplinary Integrated Center for Research of the Dermatological Interface (MIC-DIR), “Dunărea de Jos” University of Galati, 800008 Galati, Romania; 6 “Grigore T. Popa” University of Medicine and Pharmacy, 16 Universității Street, 700115 Iași, Romania; emil.anton@yahoo.com (E.A.); anca_ign@yahoo.com (A.L.); 7 Research Centre in the Medical-Pharmaceutical Field, Faculty of Medicine and Pharmacy, “Dunărea de Jos” University of Galati, 35 Al. I. Cuza Street, 800010 Galati, Romania; 8 “Sf. Ioan” Emergency Clinical Hospital for Children, 800487 Galati, Romania; 9 Faculty of Medicine and Pharmacy, “Ovidius” University of Constanța, 900470 Constanța, Romania; salimcamer@yahoo.com

**Keywords:** newborn hearing screening, adolescent mothers, neonatal risk factors, otoacoustic emissions

## Abstract

Universal newborn hearing screening is essential for early identification of sensorineural hearing loss. Infants born to adolescent mothers may be more vulnerable to abnormal screening outcomes due to biological, socio-economic, and obstetrical risk factors frequently associated with adolescent pregnancy. This study evaluates hearing screening outcomes in newborns of adolescent mothers and examines whether maternal and neonatal vulnerabilities contribute to abnormal (REFER) results. A retrospective observational study was conducted over four years (January 2021–January 2025) at the “Sf. Ap. Andrei” County Emergency Clinical Hospital, Galați, Romania. The study included 187 newborns of adolescent mothers (≤18 years) and 3203 newborns of mothers aged >19 years. All infants underwent transient evoked otoacoustic emission (TEOAE) testing within 48–72 h after birth, according to institutional protocol. PASS/REFER outcomes were recorded, and retesting was performed when indicated. Although otological conditions such as middle ear dysfunction may influence OAE responses, routine otoscopic examination and clinical assessment were performed prior to testing. Automated auditory brainstem response (AABR) testing was not routinely applied due to equipment availability and local screening practices. The final REFER rate was slightly higher in the adolescent group (5.3%) compared with the adult group (4.8%). Maternal age alone was not directly associated with abnormal outcomes; however, maternal anemia, limited prenatal care, rural residence, prematurity, and low birth weight were more frequently observed among cases with persistent REFER results. Infants born to adolescent mothers show a modestly increased likelihood of abnormal hearing screening outcomes, primarily related to cumulative maternal and neonatal vulnerabilities. Strengthening prenatal care and targeted audiological follow-up may improve early detection of sensorineural hearing loss in this population.

## 1. Introduction

Otoacoustic emissions (OAE) and automated auditory brainstem response (AABR) are the most widely used techniques in universal newborn hearing screening (UNHS) programs. OAE testing, particularly transient evoked otoacoustic emissions (TEOAE), is frequently employed as a first-line screening tool due to its non-invasive nature, rapid administration, and high sensitivity for detecting cochlear outer hair cell dysfunction [1,2]. However, OAE results may be influenced by transient middle ear conditions, such as residual amniotic fluid, vernix caseosa, or mild conductive alterations. Transient conductive conditions affecting the external or middle ear have been shown to significantly influence initial OAE screening outcomes, particularly in the early neonatal period [3]. Despite these limitations, OAE-based screening remains a practical and effective approach in many maternity settings, especially when applied within a standardized clinical protocol and followed by appropriate retesting procedures. In Romania, newborn hearing screening is implemented heterogeneously across maternity units, with OAE-based protocols being the most frequently used methods [4,5,6].

The prevalence of permanent sensorineural hearing loss (SNHL) in the general neonatal population is estimated at approximately 1–2 per 1000 live births [7], with higher rates reported among infants exposed to medical, biological, or socio-economic vulnerabilities. Prematurity, low birth weight, neonatal hypoxia, hyperbilirubinemia, intrauterine and perinatal infections, and admission to neonatal intensive care units are well-established risk factors associated with abnormal hearing screening outcomes and subsequent hearing impairment. Several studies have demonstrated that prematurity and low birth weight are associated with increased rates of abnormal otoacoustic emission results and subsequent hearing impairment [8,9]. In addition to these neonatal factors, maternal health conditions and the quality of prenatal care play a critical role in fetal auditory development and perinatal adaptation.

Adolescent pregnancy remains a significant public health concern worldwide and is frequently associated with increased maternal and neonatal morbidity. Previous population-based studies and systematic reviews have consistently reported higher rates of prematurity, low birth weight, and neonatal morbidity among infants born to adolescent mothers [10]. Adolescent mothers often face a combination of biological immaturity, limited access to prenatal healthcare, lower health literacy, and socio-economic disadvantage. These factors contribute to higher rates of obstetric complications, including anemia, infections, hypertensive disorders of pregnancy, and inadequate gestational monitoring. Consequently, infants born to adolescent mothers are more likely to be premature, have low birth weight, or experience early neonatal adaptation difficulties, all of which may increase vulnerability to abnormal hearing screening outcomes.

Beyond biological factors, social determinants of health play an important role in shaping perinatal outcomes among adolescent mothers. Rural residence, reduced family support, and delayed engagement with healthcare services may negatively influence both prenatal monitoring and postnatal follow-up. Previous studies have shown that socially vulnerable populations demonstrate lower adherence to newborn screening programs and higher rates of loss to follow-up, potentially delaying the diagnosis of sensorineural hearing loss even when screening is initially performed [11]. Socioeconomic disadvantage, rural residence, and limited access to healthcare services have been associated with increased loss to follow-up and delayed diagnosis of hearing impairment [12]. Population-based studies have also suggested an association between maternal age and the risk of adverse neonatal auditory outcomes, including failed newborn otoacoustic emissions (OAE) hearing screening [13]. Socioeconomic disadvantage has also been shown to influence newborn otoacoustic emissions (OAE) hearing screening outcomes and follow-up adherence [13]. These disparities highlight the importance of understanding not only the biological mechanisms underlying hearing screening failure but also the broader socio-medical context in which adolescent pregnancy occurs.

Although numerous studies have examined obstetric and neonatal outcomes associated with adolescent pregnancy, relatively little attention has been paid to auditory outcomes in this population. Existing research has largely focused on general neonatal morbidity, while the specific relationship between adolescent maternal age, associated vulnerabilities, and newborn hearing screening results remains insufficiently explored. In particular, it is unclear whether adolescent maternal age itself represents an independent risk factor for abnormal screening outcomes, or whether observed differences are primarily driven by the cumulative effect of medical and socio-economic vulnerabilities.

In Romania, newborn hearing screening is implemented in many maternity hospitals; however, a fully standardized national UNHS program has not yet been universally established. As a result, disparities in screening practices, follow-up, and early intervention persist, particularly among vulnerable populations. Understanding how adolescent maternal vulnerabilities influence hearing screening outcomes is therefore essential for optimizing screening protocols, improving follow-up strategies, and reducing inequalities in early hearing detection and intervention.

Against this background, the present study aims to evaluate newborn hearing screening outcomes in infants born to adolescent mothers and to assess whether maternal and neonatal vulnerabilities associated with adolescent pregnancy contribute to abnormal (REFER) screening results. By comparing outcomes between newborns of adolescent mothers and those born to adult mothers within the same institutional setting, this study seeks to clarify the extent to which biological and socio-medical factors, rather than maternal age alone, influence early auditory screening outcomes. Such insights may inform targeted surveillance strategies and support the development of more inclusive and effective newborn hearing screening programs for vulnerable maternal populations. Only a limited number of studies have specifically addressed hearing screening outcomes in infants born to adolescent mothers, highlighting challenges related to medical vulnerability and follow-up [13].

## 2. Materials and Methods

### 2.1. Study Design and Setting

This study was designed as a retrospective observational analysis conducted over a four-year period, from January 2021 to January 2025, in the Neonatology Department of the “Sf. Ap. Andrei” County Emergency Clinical Hospital, Galați, Romania. The hospital is a tertiary-level maternity unit that provides care for both low-risk and high-risk pregnancies and serves a heterogeneous population from urban and rural areas. During the entire study period, the institution applied a standardized newborn hearing screening protocol, allowing consistent data collection and comparison between study groups.

### 2.2. Study Population

The study population included all live-born infants delivered at the study center during the specified period who underwent newborn hearing screening and had complete clinical documentation available. Newborns were divided into two groups based on maternal age at delivery. The adolescent group consisted of infants born to mothers aged 18 years or younger, resulting in a total of 187 newborns. The comparison group included 3203 newborns born to mothers aged over 19 years. Infants born to mothers aged exactly 19 years were excluded to ensure a clear distinction between adolescent and adult maternal profiles.

Exclusion criteria comprised major congenital malformations, known genetic syndromes, or conditions incompatible with life, as these factors could independently influence hearing screening outcomes. Newborns with incomplete screening data or cases in which parents declined participation in the hearing screening program were also excluded from the analysis.

### 2.3. Hearing Screening Protocol

**Figure 1:**
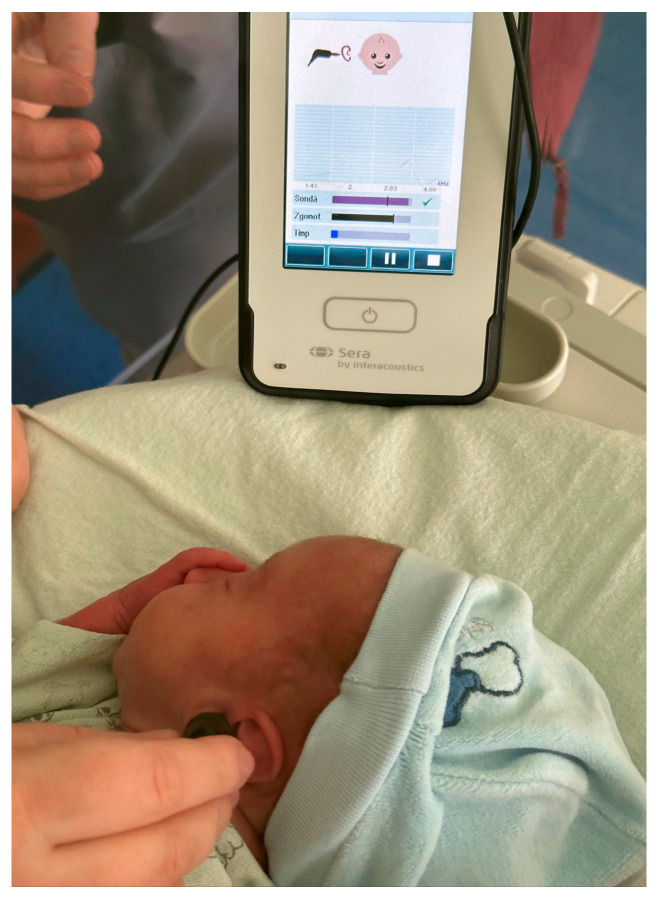
Newborn hearing screening performed using transient evoked otoacoustic emissions (TEOAE) with the SERA OAE system (Interacoustics, Middelfart, Denmark). The image illustrates the placement of the probe in the external auditory canal during routine neonatal screening.

All newborns underwent hearing screening within the first 48–72 h of life, in accordance with institutional protocol and international recommendations. Transient evoked otoacoustic emissions (TEOAE) were recorded using the SERA OAE system, a device commonly used for neonatal hearing screening (Figure 1).

The screening protocol involved acoustic stimulation delivered at an intensity of approximately 80 dB SPL, with responses recorded across frequencies ranging from 1 to 5 kHz. A signal-to-noise ratio (SNR) of at least 6 dB was required for a valid response at each tested frequency. Reproducibility criteria were applied automatically by the device software to ensure response reliability. A screening result was classified as PASS when otoacoustic emissions were present and met all predefined criteria in both ears. A REFER result was assigned when emissions were absent or insufficient in one or both ears.

Given that OAE measurements may be influenced by transient middle ear conditions, routine clinical assessment was performed prior to testing. This included otoscopic examination and evaluation of the external auditory canal to identify visible obstructions or otological abnormalities. Tympanometry was not routinely performed as part of the screening protocol due to equipment availability and the retrospective nature of the study. Automated auditory brainstem response (AABR) testing was not systematically applied, as TEOAE constituted the standard first-line screening method at the study center during the study period.

Newborns who initially failed the screening underwent repeat TEOAE testing prior to discharge whenever possible. The final PASS or REFER classification was based on the outcome after retesting.

### 2.4. Maternal and Neonatal Data Collection

Maternal data were extracted from electronic medical records and included maternal age, place of residence (urban or rural), adherence to prenatal care, and the presence of medical or obstetric conditions commonly associated with adolescent pregnancy. These conditions included iron-deficiency anemia, urinary tract infections, hypertensive disorders of pregnancy, gestational diabetes, and obstetric complications documented during pregnancy or delivery.

Neonatal data collected for analysis included gestational age at birth, birth weight, Apgar scores, mode of delivery, early neonatal adaptation, and admission to neonatal intensive care when applicable. Prematurity and low birth weight were specifically recorded, given their established association with abnormal hearing screening outcomes.

### 2.5. Ethical Considerations

The study was conducted in accordance with the principles outlined in the Declaration of Helsinki. Ethical approval for the retrospective analysis of anonymized clinical data was granted by the Ethics Committee of the “Sf. Ap. Andrei” County Emergency Clinical Hospital, Galați. Written informed consent was obtained from all mothers for newborn hearing screening as part of routine clinical care. For the purposes of this retrospective study, all data were fully anonymized, and no additional informed consent was required in accordance with local regulations and institutional ethical approval.

### 2.6. Statistical Analysis

Data were analyzed using descriptive and comparative statistical methods. Continuous variables were summarized using means and standard deviations or medians and interquartile ranges, as appropriate. Categorical variables were expressed as absolute numbers and percentages. Hearing screening outcomes were compared between groups using a z-test for the comparison of two independent proportions. A *p*-value of less than 0.05 was considered statistically significant. Statistical analyses were performed to evaluate differences in final REFER rates and to explore associations between maternal and neonatal vulnerabilities and abnormal screening outcomes.

## 3. Results

### 3.1. Maternal Characteristics

A total of 3390 newborns were included in the analysis, of whom 187 were born to adolescent mothers aged 18 years or younger and 3203 were born to mothers aged over 19 years. Clear differences were observed between the two maternal groups in terms of medical and socio-demographic characteristics. Adolescent mothers were more frequently from rural areas and demonstrated lower adherence to recommended prenatal care visits compared with adult mothers. In addition, maternal conditions commonly associated with adolescent pregnancy, such as iron-deficiency anemia and recurrent urinary tract infections, were more prevalent in the adolescent group.

Obstetric complications, including threatened preterm labor and the need for cesarean delivery, were also observed more frequently among adolescent mothers. These findings reflect the cumulative biological and socio-medical vulnerabilities associated with adolescent pregnancy and provide important context for the interpretation of neonatal outcomes and hearing screening results.

### 3.2. Neonatal Characteristics

Newborns born to adolescent mothers exhibited a higher prevalence of prematurity and low birth weight compared with those born to adult mothers. Although most infants had favorable Apgar scores and adapted well in the immediate postnatal period, mild respiratory distress and feeding difficulties were more commonly documented in the adolescent-mother group. Admission to neonatal intensive care units occurred more frequently among newborns of adolescent mothers, reflecting a higher burden of perinatal vulnerability.

Gestational age and birth weight emerged as key neonatal variables associated with abnormal hearing screening outcomes. Newborns who ultimately received a REFER result were more likely to be late preterm or to have low birth weight, regardless of maternal age group. These findings underscore the importance of considering neonatal maturity and early adaptation when interpreting otoacoustic emission screening results.

### 3.3. Hearing Screening Outcomes

**Figure 2:**
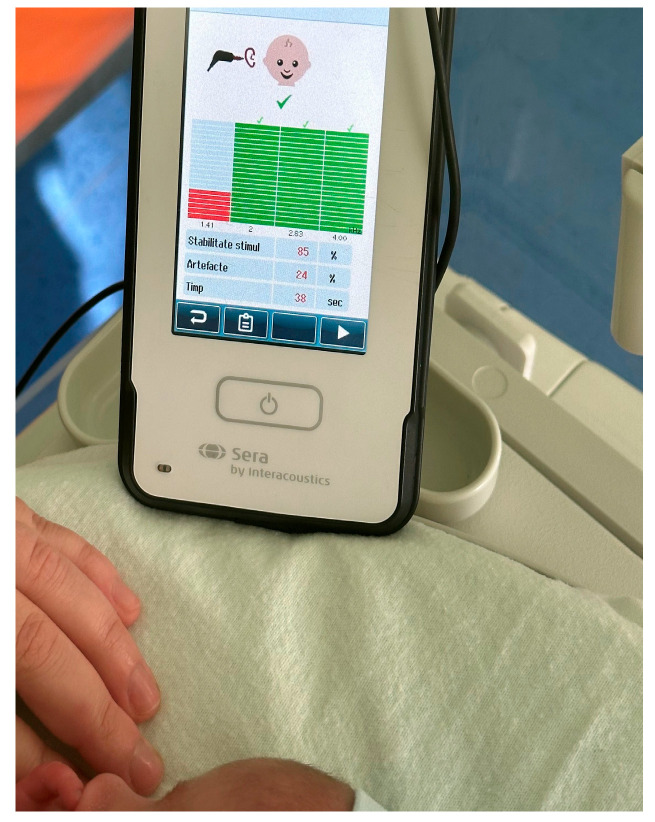
Representative example of a PASS transient evoked otoacoustic emission (TEOAE) response obtained during neonatal hearing screening, illustrating reproducible emissions across the tested frequency range.

**Figure 3:**
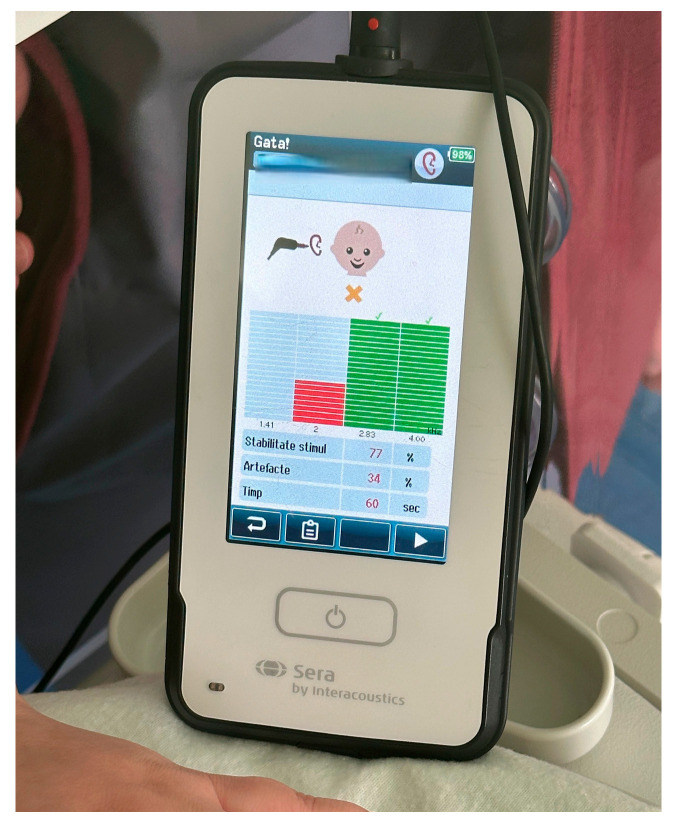
Representative example of a REFER otoacoustic emission response.

Hearing screening outcomes for both study groups are summarized in Table 1. Representative examples of transient evoked otoacoustic emission (TEOAE) screening outcomes are provided in the Supplementary Materials. A typical PASS response, characterized by reproducible emissions across the tested frequency range, is illustrated in Figure 2, while an example of a REFER response with absent or insufficient otoacoustic emissions is shown in Figure 3.

In the adolescent-mother group, 11 of the 187 newborns (5.9%) initially failed the TEOAE screening. After repeat testing, one infant achieved a PASS result, resulting in a final REFER rate of 5.3% (10/187). In the adult-mother group, 233 of the 3203 newborns (7.3%) initially received a REFER result. Following retesting, 79 newborns passed, while 154 remained REFER, yielding a final REFER rate of 4.8% (154/3203).

Although the final REFER rate was slightly higher among newborns of adolescent mothers, statistical comparison using a z-test for independent proportions revealed no significant difference between groups (z ≈ 0.33, *p* = 0.738). The pooled proportion was calculated as *p* = 0.0484, reflecting the overall frequency of abnormal screening outcomes in the study population.

At a 90% confidence level, the margin of error was wider in the adolescent group (2.65%) compared with the adult group (0.75%), a finding attributable to the smaller sample size of the adolescent cohort. This difference highlights the limited statistical power to detect small between-group differences and underscores the need for cautious interpretation of the observed trend.

### 3.4. Association Between Maternal and Neonatal Vulnerabilities and REFER Outcomes

Further descriptive analysis indicated that persistent REFER outcomes were more frequently associated with specific maternal and neonatal vulnerabilities rather than maternal age alone. Among newborns with final REFER results in the adolescent group, maternal anemia, limited prenatal care, rural residence, and documented infections during pregnancy were more commonly observed. From a neonatal perspective, prematurity and low birth weight were the most frequently identified characteristics among infants with abnormal screening outcomes.

These findings suggest that the increased proportion of REFER results observed in the adolescent-mother group may be driven by the cumulative effect of biological and socio-medical risk factors. The coexistence of maternal vulnerabilities and neonatal immaturity appears to contribute to an increased likelihood of abnormal TEOAE responses, supporting a multifactorial interpretation of newborn hearing screening failure.

To determine whether the difference between the two groups was statistically significant, a z-test for the comparison of two independent proportions was applied. The final REFER rate was 5.35% (10/187) in the adolescent group and 4.81% (154/3203) in the adult group. The pooled proportion was *p* = 0.0484. The z statistic was calculated using:

z = (p_1_ − p_2_)/√[p(1 − p) × (1/n_1_ + 1/n_2_)]

with p_1_ = 0.0535, p_2_ = 0.0481, p = 0.0484, n_1_ = 187, and n_2_ = 3203. 

The resulting value was z ≈ 0.33, with a two-tailed *p*-value of 0.738, indicating no statistically significant difference (*p* > 0.05). Although the difference in final REFER rates between groups was modest, further analysis suggests that the higher proportion observed among newborns from adolescent mothers may be influenced by the vulnerabilities characteristic of adolescent pregnancy. Newborns with persistent REFER outcomes were more frequently associated with maternal anemia, limited prenatal care, recurrent infections, rural residence, and neonatal factors such as prematurity or low birth weight. These findings indicate that maternal and neonatal vulnerabilities—rather than maternal age alone—may contribute to an increased likelihood of abnormal newborn hearing screening results in infants of adolescent mothers.

## 4. Discussion

The findings of the present study indicate that newborns of adolescent mothers show a slightly higher rate of abnormal newborn hearing screening (final REFER) compared with those born to adult mothers. Although the difference did not reach statistical significance, the observed trend is consistent with previous reports describing adolescent pregnancy as a condition associated with increased maternal and neonatal vulnerability [14].

Adolescent mothers in our cohort presented higher frequencies of medical and obstetric risk factors, such as iron-deficiency anemia, recurrent urinary tract infections, threatened preterm labor, and cesarean delivery. These conditions are well-documented contributors to adverse neonatal outcomes and may indirectly influence auditory function. Previous studies have demonstrated that prematurity, low birth weight, neonatal respiratory distress, and delayed postnatal adaptation are associated with a higher probability of abnormal otoacoustic emission responses or subsequent hearing impairment [15].

Although maternal age alone was not identified as a statistically significant predictor of hearing screening outcomes in the present study, the coexistence of multiple biological and socio-medical vulnerabilities suggests a multifactorial mechanism. This observation supports the concept that adolescent pregnancy represents not only a biological risk factor but also a complex vulnerability cluster shaped by limited prenatal care, reduced health literacy, and socio-economic disadvantage [16].

Placental dysfunction may represent an important upstream biological mechanism linking maternal vulnerabilities to adverse neonatal outcomes. Increasing evidence highlights the role of angiogenic imbalance—particularly alterations in the soluble fms-like tyrosine kinase-1 (sFlt-1) and placental growth factor (PlGF) ratio—in the pathophysiology of preeclampsia and placental insufficiency. Ciciu et al. demonstrated that the sFlt-1/PlGF ratio is a valuable biomarker for adequate preeclampsia management, reflecting the severity of placental dysfunction and its systemic impact on maternal and fetal health [17].

Although hypertensive disorders of pregnancy and angiogenic biomarkers were not specifically assessed in the present study, placental insufficiency remains highly relevant in the context of adolescent pregnancy. Adolescents are known to exhibit higher rates of inadequate prenatal care, nutritional deficiencies, anemia, and untreated infections—factors that may exacerbate placental stress and vascular dysfunction. Impaired placental function has been strongly associated with prematurity, low birth weight, and intrauterine growth restriction, which are recognized risk factors for abnormal otoacoustic emission responses and subsequent hearing impairment [8,9].

These findings reinforce the multifactorial nature of newborn hearing screening failure. Rather than maternal age acting as an isolated determinant, the cumulative burden of biological, obstetric, and socio-economic vulnerabilities appears to play a more substantial role. In infants born to adolescent mothers, the convergence of placental dysfunction, prematurity, low birth weight, and delayed neonatal adaptation may increase susceptibility to transient or persistent cochlear dysfunction, resulting in REFER outcomes during early screening [18].

From a clinical and public health perspective, these results underscore the importance of enhanced surveillance strategies for newborns born to adolescent mothers. Addressing disparities in early hearing detection requires targeted strategies for vulnerable maternal populations, as emphasized by global public health initiatives [19]. Even when the initial hearing screening result is normal, this population may benefit from closer audiological follow-up due to the higher prevalence of underlying risk factors.

The present study has several strengths, including a four-year observational period, standardized hearing screening protocols, and a comparison group derived from the same institutional setting. However, certain limitations must be acknowledged. The relatively small size of the adolescent cohort may have limited the statistical power to detect significant differences between groups. Additionally, the absence of automated auditory brainstem response testing, tympanometry, placental biomarkers, and long-term audiological follow-up limited the depth of mechanistic interpretation.

From a public health perspective, our findings highlight the need for strengthened hearing follow-up protocols among newborns of adolescent mothers—even when the initial screening is passed or when abnormal results do not reach statistical significance. Early detection is critical, and newborns from medically or socially vulnerable populations require systematic surveillance.

Furthermore, the absence of statistical significance should not obscure potential clinical relevance. The small sample size of the adolescent group (n = 187) and the consequent wider margin of error (2.65%) may reduce the power to detect meaningful differences. Larger studies or multicenter analyses may provide more robust evidence on whether adolescent maternal age, independent of associated vulnerabilities, contributes to neonatal auditory risk.

Overall, the results reinforce the importance of integrating audiological follow-up into comprehensive care programs for adolescent mothers. Such strategies may help mitigate disparities in early hearing detection and intervention and contribute to improved long-term neurodevelopmental outcomes.

Despite the relevance of the findings, several limitations of the present study should be acknowledged. First, the retrospective observational design inherently limits the ability to establish causal relationships between maternal age, associated vulnerabilities, and abnormal newborn hearing screening outcomes. Second, the relatively small size of the adolescent-mother cohort compared with the adult-mother group may have reduced the statistical power to detect subtle but potentially clinically meaningful differences in REFER rates.

Third, hearing screening was based exclusively on transient evoked otoacoustic emissions, which, although widely used as a first-line screening tool, may be influenced by transient middle ear conditions and do not assess neural auditory pathway integrity. Automated auditory brainstem response testing and tympanometry were not routinely available during the study period, limiting a more comprehensive audiological evaluation. In addition, detailed data regarding hypertensive disorders of pregnancy, placental biomarkers, and long-term audiological follow-up were not available, precluding further exploration of underlying biological mechanisms and the persistence of hearing impairment beyond the neonatal period.

Finally, socio-economic variables were assessed indirectly through clinical records and place of residence, which may not fully capture the complexity of social determinants influencing prenatal care and postnatal follow-up. Future prospective, multicenter studies incorporating comprehensive audiological assessments, placental and perinatal biomarkers, and long-term follow-up are warranted to better elucidate the relationship between adolescent maternal vulnerability and neonatal hearing outcomes.

## 5. Conclusions

The results of this study suggest that newborns of adolescent mothers may present a slightly higher rate of abnormal auditory screening outcomes compared with those of adult mothers, although the difference was not statistically significant. This pattern appears to be influenced not by maternal age alone, but by the cumulative effect of multiple vulnerabilities—medical, obstetric, and socio-economic—more frequently observed among adolescent mothers.

Given these findings, newborns from this population should be considered a clinically important group for enhanced audiological surveillance. Even when the initial hearing screening is passed, or when abnormal results do not demonstrate statistical significance, systematic follow-up may help ensure early detection of potential auditory deficits and improve long-term neurodevelopmental outcomes.

Future research using larger, multicenter cohorts is needed to further clarify the relationship between adolescent maternal age and neonatal hearing outcomes, and to determine whether targeted interventions can reduce disparities in early hearing detection and intervention for this vulnerable population.

## Figures and Tables

**Table 1:** Hearing screening outcomes in newborns from adolescent and adult mothers. Note: REFER indicates failure of otoacoustic emission screening; percentages are calculated within each maternal age group.

Maternal Group	Total Newborns	Initial REFER	Final REFER	Final REFER Rate
**Mothers ≤ 18 years**	187	11	10	5.30%
**Mothers > 19 years**	3203	233	154	4.80%

## Data Availability

The data supporting the findings of this study are available from the corresponding authors upon reasonable request. The dataset is not publicly available due to ethical and privacy restrictions involving patient medical records.

## Abbreviations

The following abbreviations are used in this manuscript:

OAE	Otoacoustic Emissions
TEOAE	Transient Evoked Otoacoustic Emissions
ABR	Auditory Brainstem Response
NICU	Neonatal Intensive Care Unit
PASS	Normal response on newborn hearing screening
REFER	Abnormal response on newborn hearing screening
dB	Decibel
SNR	Signal-to-Noise Ratio
WHO	World Health Organization
JCIH	Joint Committee on Infant Hearing
IRB	Institutional Review Board
CI	Confidence Interval
RR	Relative Risk
LBW	Low Birth Weight
GA	Gestational Age
APC	Article Processing Charge
